# A Two-Phase Coverage-Enhancing Algorithm for Hybrid Wireless Sensor Networks

**DOI:** 10.3390/s17010117

**Published:** 2017-01-09

**Authors:** Qingguo Zhang, Mable P. Fok

**Affiliations:** 1 College of Computer, Huazhong Normal University, Wuhan 430079, China; 2 Lightwave and Microwave Photonics Laboratory, College of Engineering, University of Georgia, Athens, GA 30602, USA; mfok@uga.edu

**Keywords:** hybrid wireless sensor network, differential evolution, area coverage, mobile sensor, static sensor

## Abstract

Providing field coverage is a key task in many sensor network applications. In certain scenarios, the sensor field may have coverage holes due to random initial deployment of sensors; thus, the desired level of coverage cannot be achieved. A hybrid wireless sensor network is a cost-effective solution to this problem, which is achieved by repositioning a portion of the mobile sensors in the network to meet the network coverage requirement. This paper investigates how to redeploy mobile sensor nodes to improve network coverage in hybrid wireless sensor networks. We propose a two-phase coverage-enhancing algorithm for hybrid wireless sensor networks. In phase one, we use a differential evolution algorithm to compute the candidate’s target positions in the mobile sensor nodes that could potentially improve coverage. In the second phase, we use an optimization scheme on the candidate’s target positions calculated from phase one to reduce the accumulated potential moving distance of mobile sensors, such that the exact mobile sensor nodes that need to be moved as well as their final target positions can be determined. Experimental results show that the proposed algorithm provided significant improvement in terms of area coverage rate, average moving distance, area coverage–distance rate and the number of moved mobile sensors, when compare with other approaches.

## 1. Introduction

Wireless sensor networks (WSNs) are composed of a large number of sensor nodes that have limited resources such as energy, bandwidth, memory, and processing power. WSNs are widely used in many important applications such as environmental monitoring, disaster management, traffic analysis, and intrusion detection. One key objective of these applications is to monitor a field of interest to detect movement, temperature changes, precipitation, and so forth, which depends on the coverage quality of the sensor network. Coverage is usually defined as a measure of how well and how long the sensors are able to observe the physical space. The quality of coverage in static sensor is significantly affected by the initial deployment location of the sensors. Unfortunately, sensor deployment cannot be performed manually in most applications due to the remote or hostile working environments of WSNs. Thus, sensors are usually deployed by scattering them from an aircraft; however, the actual landing position cannot be controlled due to the existence of wind and obstacles such as trees and buildings. Consequently, some subareas may not have sufficient sensor coverage no matter how many sensors are dropped, and some subareas may even have coverage holes (i.e., areas that are not covered by any sensor node). Therefore, even if a large number of redundant nodes are deployed, the desired level of coverage still cannot be achieved, not to mention the high cost that is associated with the redundant nodes. 

One solution to the above problem is to enhance part of the sensors with mobility. The recent advancements of embedded hardware and miniaturized robotics have made mobile sensors possible. Mobile sensors have the same sensing capability as static sensors and they are able to move to the correct locations for providing the required coverage after the initial deployment. However, equipping every sensor with mobile capability will increase the sensor network cost, which also makes routing and information exchange become very complicated. On the other hand, a hybrid wireless sensor network assisted by a small set of mobile sensors could be a cost-effective solution towards improving coverage with unevenly deployed sensors. Hybrid sensor networks composed of mobile and static sensors open a new frontier of research in WSNs. The research focus of hybrid WSNs is increasing due to the strong general interest in mobility as a design parameter in WSNs [1]. Applications of hybrid WSNs have been studied in [2]. In this paper, we focus on how to redeploy mobile sensor nodes to improve network coverage in hybrid WSNs. 

The most important coverage problems can be classified into the following three types: area coverage, point coverage, and barrier coverage. This paper focuses on the area coverage problem, where the main objective of the sensor network is to monitor an area and to solve the following area coverage problem in hybrid WSNs: Given a randomly deployed hybrid WSN with all sensors knowing their locations on a plane, determine where the mobile sensors should be moved such that the area coverage can be maximized, while incurring the least moving cost. The goal is to minimize the moving cost including the accumulated moving distance, total number of moves, and communication/computation cost. Generally, in order to get higher coverage in hybrid WSNs, a large number of mobile nodes need to move, resulting in a significant increase in moving cost and an increase in energy consumption. Therefore, how to balance network coverage and moving cost of mobile sensors is a challenging question. 

In this paper, we present a two-phase network coverage-enhancing algorithm for WSNs. The algorithm aims to achieve a balance between network coverage and the overall mobile sensor moving distance by a well-designed objective function of them, resulting in a high coverage rate but with a short moving distance. This paper is organized as follows: we first discuss existing approaches for WSN coverage optimization in Section 2, then we describe our proposed two-phase network coverage-enhancing algorithm in Section 3. Performance of the proposed algorithm is evaluated in Section 4. Lastly, we summarize our findings in Section 5.

## 2. Related Work

Hybrid WSNs have been widely studied in recent years due to the low cost associated with mobile sensors production, as well as the practical issue that hinder the deployment of fully mobile sensor network. For example, when all the mobile sensors are in random movement in a fully mobile sensor network, packet routing and information dissemination will be too complicated to be practical [3]. Effective coverage is one of the key problems in sensor networks, and can be applied to various applications like localization problems, where sensors are deployed to achieve minimum estimation error [4]. Moving mobile sensors to meet network coverage requirement in hybrid WSNs has received a lot attention recently. The first study towards building a hybrid WSN [5] compensates poor initial sensor distributions by strategically repositioning part of the mobile sensors. Existing sensor relocation schemes are classified into three types [6]: coverage pattern-based movement [7,8], virtual force-based movement [9,10,11,12], and grid quorum-based movement [13,14,15,16,17,18,19]. The main idea of the coverage pattern moving scheme is to use polygons to tile-up the whole sensor field, and the vertices of these polygons are the target locations for mobile nodes. Virtual force movement schemes use repulsive and attractive forces to make nodes evenly distributed. More specifically, if the distance between two sensors is below the threshold, there is a repulsive (negative) force to push them apart; otherwise, there is an attractive (positive) force to attract them to one another. Grid architecture-based schemes partition the sensor area into a number of small grids. A grid is regarded as being covered by a sensor if the center point of the square grid lies within the sensor’s sensing area, and the whole sensor area is regarded as being covered if all the grids are covered. 

In order to meet coverage requirements, many movement algorithms have been proposed in literature based on grid architecture. Guiling et al. [13] explored the motion capability of sensors for relocation to deal with sensor failure or respond to new events. They proposed a two-phase sensor relocation solution: redundant sensors are first identified and then relocated to the target location. They proposed a grid-quorum solution to locate the closest redundant sensor, and proposed to use cascaded movement to relocate the redundant sensor. In order to meet coverage and load balancing requirements, Jie and Shuhui [14] focus on minimizing the total moving distance and propose an optimal, but centralized, movement solution, based on the Hungarian method. However, the algorithms in [13,14] assumed that all sensors are mobile, and the entire sensors have the capability to move to any grid in the area, which is impractical. Our algorithm assumes that only a small portion of sensors that can move. 

Since the coverage problem in WSNs is nondeterministic polynomial time (NP)-complete [20,21], a number of researchers have attempted to use intelligent algorithms such as genetic algorithm (GA) and particle swarm optimization (PSO) to solve this problem. GAs [22,23,24] are used to maximize area coverage in WSNs. The area coverage is chosen as the objective function of the GAs. However, the above genetic algorithms can only be applied to static sensor networks. For hybrid WSNs, performances of the above genetic algorithms are not satisfactory, which we will discuss further in Section 4. The disadvantage of the objective function in [22,23,24] is that it may incur high moving costs when maximizing the area coverage in hybrid WSNs. PSO is another stochastic optimization algorithm that is used for the coverage problem in [25,26]; the major difference between GA and PSO is that PSO have no explicit selection, crossover, and mutation operations. However, the objective functions of the PSO algorithms in [25,26] have the same disadvantage as those of GA in [22,23,24].

Differential evolution (DE) is a very popular evolutionary algorithm that exhibits remarkable performance in a wide variety of problems in diverse fields. DE has proved to be the fastest evolutionary algorithm in the first International IEEE Competition on Evolutionary Optimization, and has been shown to be better than the GA or PSO in many case. This motivates us to apply DE to the coverage optimization problem in WSN. In this paper, we propose a two-phase network coverage-enhancing algorithm to solve the problem in [22,23,24,25,26]. In phase one, we use a DE algorithm to compute the candidate’s target position of each mobile sensor node. In phase two, we perform refinement by applying an optimization scheme on the candidates’ target positions calculated in phase one to reduce the accumulated moving distance of the mobile sensors. This information is then used to determine the exact mobile sensor nodes that are needed to move and the corresponding final target positions. The contributions of this paper are:

1. Propose a novel coverage-enhancing algorithm based on DE for WSNs. The algorithm can achieve a balance between network coverage and mobile sensor moving distance.
2. Propose a moving-distance optimization scheme which can reduce the number of mobile sensors needed to be moved as well as greatly reduce the overall moving cost.

## 3. The Two-Phase Coverage-Enhancing Algorithm

### 3.1. Initial Assumptions

In this paper, our discussion is built upon the following assumptions:

(1) All sensors have the same sensing range *R_s_* and communication range *R_c_* = 2*R_s_*.
(2) Each sensor knows its location by a certain mechanism, such as Global Positioning System (GPS), and the location information can be sent to the base station (BS).
(3) Mobile sensor nodes are able to move to the scheduled positions, where the scheduled positions are within their mobility range.
(4) The proposed algorithm is implemented in a centralized architecture, and the BS is responsible for the execution of the algorithm and broadcasting the movement plan of mobile sensors.

### 3.2. Problem Formulation

Assume the sensor area *A* is a two-dimensional plane, the target area is digitized into L*W grids where each grid size is equal to 1 [27]. A hybrid wireless sensor network with a total of *n* sensors is deployed on the target area, where *m* of them are mobile sensors and *n* − *m* are static sensors. The sensor node set is defined as:

*S* = {*s*_1_, *s*_2_, …, *s_m_*,…, *s_n_*} (1)

Let sensor *s_i_* be the sensor deployed at point (*x_i_*,*y_i_*). The coverage range of sensor *s_i_* can be expressed as a circle centered at its coordinates (*x_i_*,*y_i_*) with the radius of the sensing range *R_s_*. A random variable *E_i_* is introduced to describe the event that the sensor node *s_i_* covers a grid point *G*(*x_G_*,*y_G_*). The probability of event *E_i_*, denoted as *P*{*E_i_*}, is equal to the coverage probability *P*(*s_i_*,*x_G_*,*y_G_*). Therefore, the probability is described by the following Boolean sensing model [28]:

(2) $$P\left(s_{i},x_{G},y_{G}\right)=\{\begin{array}{c} 1,\;\mathrm{if}\;d\left(s_{i},x_{G},y_{G}\right)\leq R_{s}\\ 0,\;\mathrm{otherwise}\; \end{array}$$

where $\left(s_{i},x,y\right)=\sqrt{{\left(x_{i}-x\right)}^{2}+{\left(y_{i}-y\right)}^{2}}$, which is the Euclidean distance between a sensor *s_i_* and a point *G*(*x*,*y*). All points within such a disk have a coverage measure of 1, and are regarded as being covered by the particular sensor. All points outside such a disk have a coverage measure of 0, and are referred to as not being covered by that particular sensor. It is assumed that any random event *E_i_* is independent of the others, so *E_i_* and *E_j_* are not related, such that *i*, *j* ∈ [1, *n*] and *i* ≠ *j*. Thus, the following two relationships can be obtained [29].

(3) $$P\left\{\bar{E_{i}}\right\}=1-P\left\{E_{i}\right\}=1-P\left(s_{i},x,y\right)$$

(4) $$P\left\{E_{i}\cup \text{​}E_{j}\right\}=1-P\left\{\bar{E_{i}}\cap \text{​}\bar{E_{j}}\right\}=1-P\left\{\bar{E_{i}}\right\}.P\left\{\bar{E_{j}}\right\}$$

where $\bar{E_{i}}$ is the complement of *E_i_*, denoting the event that sensor node *s_i_* does not cover a grid point *G*(*x*,*y*). It can be considered that the grid point *G*(*x*,*y*) is covered by the node set if any node in the set covers it. Therefore, the probability of the event that the grid point (*x*,*y*) is covered by the node set can be considered as the union of *E_i_*, as follows:

(5) $$P\left(S,x,y\right)=P\left\{\overset{n}{\underset{i=1}{\cup }}E_{i}\right\}=1-P\left\{\overset{n}{\underset{i=1}{\cap }}\bar{E_{i}}\right\}=1-\overset{n}{\underset{i=1}{\prod }}\left(1-P\left(s_{i},x,y\right)\right)$$

Next, we define area coverage rate and area coverage–distance rate, which are used as metrics for performance evaluation of coverage control algorithms.

**Definition 1** (Area coverage rate)**.** *The regional coverage rate of a sensor node set S is defined as R_area_(S):*

(6) $$R_{area}\left(S\right)=\frac{A_{area}\left(S\right)}{A}=\frac{\sum_{x=1}^{L}\sum_{y=1}^{w}P\left(S,x,y\right)}{L*W}$$

*where A_area_(S) is the covering area of the sensor node set S, and A is the total area of the target region. A high area coverage rate ensures a high quality of service provided by the WSN. Obviously, the larger R_area_(S) is, the better the quality of coverage is. So, Equation (6) is selected as the object function of many coverage optimization algorithms based on intelligent algorithms [24,25,26] by maximizing R_area_(S).*

**Definition 2** (Area coverage–distance rate)**.** *Area coverage–distance rate is another useful metric to evaluate the performance of a coverage optimization algorithm. The area coverage–distance rate RD(S) of a sensor node set S is defined as:*

(7) $$RD\left(S\right)=\frac{R_{area}\left(S\right)}{average\_distance\left(S\right)}$$

*where average_distance(S) is the average moving distance of the mobile sensors. The area coverage–distance rate reflects the “efficiency” of the moving distance, which corresponds to the rate of change of area coverage with respect to the moving distance of mobile sensors. That is to say, the larger the value of RD(S), the better. In order to increase the value of RD(S), we should increase the numerator R_area_(S) and reduce the denominator average_distance(S). Therefore, we use R_area_(S), the regional coverage rate of a sensor node, and average_distance(S), the average moving distance of the mobile sensors, as the two optimization objectives of our algorithm, while having the area coverage–distance rate equation (described in Equation (7)) as the object function of our algorithm. In general, an increase in area coverage is usually accompanied by an increase of the average moving distance. When the balance between the area coverage and average moving distance is achieved, the value of RD(S) will increase. On the other hand, reducing the average moving distance of mobile sensor nodes while keeping the area coverage unchanged can also increase the value of RD(S), which is what our algorithm does in its second phase.*

### 3.3. Phase I: The Coverage-Enhancing Algorithm Based on Differential Evolution (CEADE)

Our approach utilizes a coverage-enhancing algorithm based on differential evolution (DE). DE [30] is a stochastic search algorithm, which exploits a population of potential solutions, individuals, to probe the search space. DE has the advantage of being easy to understand, simple to implement, and easy to work with, so that DE can be used for a wide variety of design and optimization tasks.

#### 3.3.1. Coding and Initialization

The CEADE algorithm uses the following real number vector to denote the solution to the coverage problem:

*X_i_* = (*x_i_*_1_, …, *x_ii_*, …, *x_iD_*) (8)

where 1 ≤ *i* ≤ NP, with NP as the size of the population; *x_ii_* ∈ [*a_i_*, *b_i_*], with *a_i_* and *b_i_* as the lower bound and upper bound of the *i*th gene, respectively; *D* = 2*m*, with *m* as the number of mobile nodes. All the NP individuals are randomly generated in the D-dimension space.

#### 3.3.2. Mutation

In each generation *t*, the mutation operation is applied to the best vector to create its corresponding mutant vector. The mutant vector is generated according to the following equation:

(9) $$v_{i}\left(t+1\right)=X_{best}+F\left(X_{r2}\left(t\right)-X_{r3}\left(t\right)\right)+F\left(X_{r4}\left(t\right)-X_{r5}\left(t\right)\right)$$

where *t* denotes the current generation, *X_best_* represents the vector with the best fitness value in current generation, *r*_2_, *r*_3_, *r*_4_, and *r*_5_ are random integers, *r*_2_, *r*_3_, *r*_4_, *r*_5_ ∈ {1, …, NP} and *i* ≠ *r*_2_ ≠ *r*_3_ ≠ *r*_4_ ≠ *r*_5_ ≠ *best. F* is *scaling factor*, which is a positive control parameter for amplifying the difference vectors. Usually the choice for the scaling factor is a constant between 0.4 and 1. 

#### 3.3.3. Crossover

In order to increase population diversity, crossover is performed. The mutant vector $v_{i}\left(t+1\right)$ is mated with $X_{i}\left(t\right)$ to generate a trial vector *u_i_*(*t* + 1). The process can be expressed as

(10) $$u_{i,j}\left(t+1\right)=\{\begin{array}{c} v_{i,j}\left(t+1\right),\;\mathrm{if}\;(rand\left[0,1\right]<CR\;\mathrm{or}\;j=j_{rand})\\ X_{i,j}\left(t\right),\;\mathrm{otherwise} \end{array}$$

where *rand*(0,1) is a uniformly distributed random number in the interval [0, 1], *j_rand_* is an integer randomly generated within the range [1, *D*] , which is used to ensure the trial vector gets at least one parameter from the mutant vector v_i_. The crossover probability *CR* is another control parameter ∈ [0, 1], which determines the fraction of vector components inherited from the mutant vector.

#### 3.3.4. Selection

To decide whether the trial vector *u_i_*(*t* + 1) should become a member of the next generation, it is compared to the target vector *X_i_*(*t*). For a maximization problem, the vector with the larger fitness value survives to the next generation, which can be expressed as follows:

(11) $$X_{i}\left(t+1\right)=\{\begin{array}{c} u_{i}\left(t+1\right)\;,\;\mathrm{if}\;\left(f\left(u_{i}\left(t+1\right)\right)\geq f\left(X_{i}\left(t\right)\right)\right)\\ X_{i}\left(t\right),\;\mathrm{otherwise} \end{array}$$

where *f*(*x*) is the objective function for the maximization problem.

#### 3.3.5. Termination Condition

The termination condition of our DE is simply checking whether the algorithm has been running for a fixed number of generations. When our DE algorithm terminates, it will output the candidate target positions for the mobile sensor nodes, which are going to be the inputs of phase two in our algorithm.

### 3.4. Phase II: Refinement

The goal for the refinement in phase two is the reduction of the moving distance of mobile sensor nodes. The refinement aims to reduce the number of mobile sensor nodes that need to move, as well as to reduce the average moving distance of mobile sensors; however, it will not reduce the network coverage. According to Equation (7), the area coverage-distance rate *RD*(*S*) of the sensor node set S will increase after phase two, which enables our algorithm to achieve good performance in terms of average moving distance, area coverage-distance rate, and the number of moved mobile sensors. The algorithm proposes that mobile sensors perform virtual movements during the process of optimization of network coverage, while physical movements will only be performed after the final destinations are identified.

**Figure 1:**
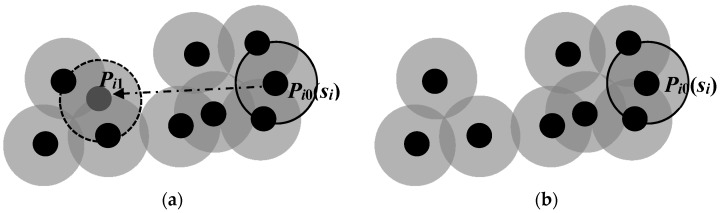
Reduce the number of mobile sensor nodes need to move. (**a**) The movement plan of Sensor *s_i_*; (**b**) Cancellation of the movement plan of *s_i_*.

**Figure 2:**
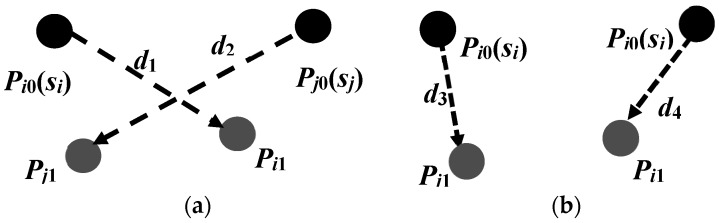
Candidate target positions exchange to reduce the average moving distance. (**a**) Before exchange; (**b**) After exchange.

**Figure 3:**
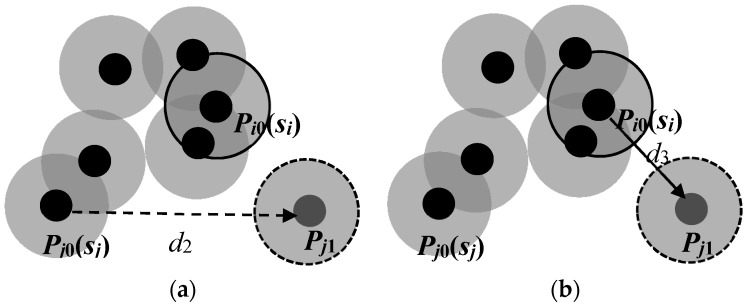
Movement replacement to reduce the average moving distance. (**a**) Sensor *s_j_* moves; (**b**) Sensor *s_i_* moves.

Let *P_i_*_0_(*x_i_*_0_,*y_i_*_0_) and *P_j_*_0_(*x_j_*_0_,*y_j_*_0_) be the initial positions of the *i*th mobile sensor *s_i_* and initial positions of the *j*th mobile sensor *s_j_*, respectively. *P_i_*_1_(*x_i_*_1_,*y_i_*_1_) and *P_j_*_1_(*x_j_*_1_,*y_j_*_1_) are the candidate target positions of *s_i_* and *s_j_*, respectively. *d*_1_ = $\left|\bar{P_{i0}P_{i1}}\right|$ is the length of the straight-line segment $\bar{P_{i0}P_{i1}}$, *d*_2_ = $\left|\bar{P_{j0}P_{j1}}\right|$, *d*_3_ = $\left|\bar{P_{i0}P_{j1}}\right|$, *d*_4_ = $\left|\bar{P_{j0}P_{i1}}\right|$. The optimization scheme for moving-distance reduction is described as follows:

1. Reduce the number of mobile sensor nodes that need to be moved. Since our DE algorithm searches for target positions of all mobile sensors simultaneously, the sensing areas of some sensors may be overlapping when they are located in their candidate target positions. The sensing areas of some mobile sensors may even be fully covered by those of the other sensors. In that case, such mobile sensors can be viewed as redundant nodes if they move to their candidate target positions, which means they do not need to move at the very beginning. As shown in Figure 1, small black solid circles represent sensor nodes and large gray solid circles represent the sensing areas of sensors. When sensor *s_i_* moves from *P_i_*_0_ to *P_i_*_1_, the regional coverage rate *R_area_*(*S*) has no change, so we can infer that sensor *s_i_* does not need to move. The number of mobile sensors that need to be moved can be reduced by this means, which can reduce the total moving distance of the mobile sensors.
2. Exchange the candidates’ target positions of two mobile sensor nodes if this can reduce the total moving distance of mobile sensors. Figure 2a shows that the total moving distance of *s_i_* and *s_j_* is *d*_1_ + *d*_2_ before exchanging the total moving distance, while Figure 2b shows that the total moving distance is *d*_3_ + *d*_4_ after exchange. Since *d*_1_ + *d*_2_ > *d*_3_ + *d*_4_, the total moving distance of *s_i_* and *s_j_* can be reduced after the exchange of the candidate target positions. Furthermore, the coverage area of the sensor network will not change after such an exchange, but the area coverage-distance rate will increase.
3. Replace the movement of the mobile sensor node that needs to move by that of a substitute mobile sensor node that does not need to move. The goal of this step is to avoid making a mobile sensor move for a long distance because moving a sensor for a long distance consumes too much energy. If the sensor is out of power shortly after it reaches the destination, this movement is wasted and another mobile sensor has to be found and relocated [13]. The condition of the replacement is that the movement can reduce the total moving distance but without reducing the coverage area of the sensor network. As shown in Figure 3a, sensor *s_i_* does not need to move, and sensor *s_j_* is planned to move from *P_j_*_0_ to *P_j_*_1_. Let A_1_ be the coverage area of the sensor network in Figure 3a after sensor *s_j_* moves from *P_j_*_0_ to *P_j_*_1_, and *A*_2_ be the coverage area of the sensor network in Figure 3b after sensor *s_i_* moves from *P_i_*_0_ to *P_j_*_1_ but *s_j_* remains static. Since *A*_2_ ≥ *A*_1_ and *d*_3_ < *d*_2_, the algorithm will make sensor *s_i_* instead of *s_j_* move to the candidate target position of sensor *s_j_* while sensor *s_j_* remains static. This replacement neither reduces the coverage area of the sensor network, nor increases the number of mobile sensors needed to move. Instead, it reduces the average moving distance of mobile sensors and thus improves the area coverage–distance rate.

The pseudocode for the moving distance reduction scheme is as follows:
**Algorithm 1.** Pseudocode for the moving distance reduction scheme.  /* *P_i_*_0_(*x_i_*_0_,*y_i_*_0_) and *P_i_*_1_(*x_i_*_1_,*y_i_*_1_) are the initial position and the candidate target position of the *i*th mobile sensor *s_i_*, *i* = 1, …, *m*. */ 1. **For**
*i* = 1 to *m* do 2.  *moved[i]* = true 3. **For**
*i* = 1 to *m* do 4.  *P = P_i_*_1_, *P_i_*_1_
*= P_i_*_0_ 5.  **If** (*R_area_(S)* reduces) *P_i_*_1_
*= P*; *moved[i]* = false; 6. **end**; 7. **For**
*i* = 1 to *m* do 8.  **For**
*j* = 1 to *m* do 9.   *d*_1_ = $\left|\bar{P_{i0}P_{i1}}\right|$, *d*_2_ = $\left|\bar{P_{j0}P_{j1}}\right|$, *d*_3_ = $\left|\bar{P_{i0}P_{j1}}\right|$, *d*_4_ = $\left|\bar{P_{j0}P_{i1}}\right|$. 10.    **If** (*i* ≠ *j*) and (*moved[i]*) and (*moved[j]*) and (*d*_1_ + *d*_2_ > *d*_3_ + *d*_4_) 11.     *P_i_*_1_↔*P_j_*_1_ 12. **end** 13. **For**
*j*: = 1 to *m* do 14.  **For**
*i*: = 1 to *m* do 15.   *d*_2_ = $\left|\bar{P_{j0}P_{j1}}\right|$, *d*_3_ = $\left|\bar{P_{i0}P_{j1}}\right|$ 16.   **if** (*moved[j]*) and (not *moved[i]*) 17.    **if** (*d*_2_ > *d*_3_) *P = P_j_*_1_*,P_i_*_1_
*= P_j_*_1_*,P_j_*_1_
*= P_j_*_0_ 18.      **if** (*R_area_(S)* reduces ) *P_j_*_1_
*= P*; *P_i_*_1_
*= P_i_*_0_ 19. **end**

## 4. Experimental Results

**Figure 4:**
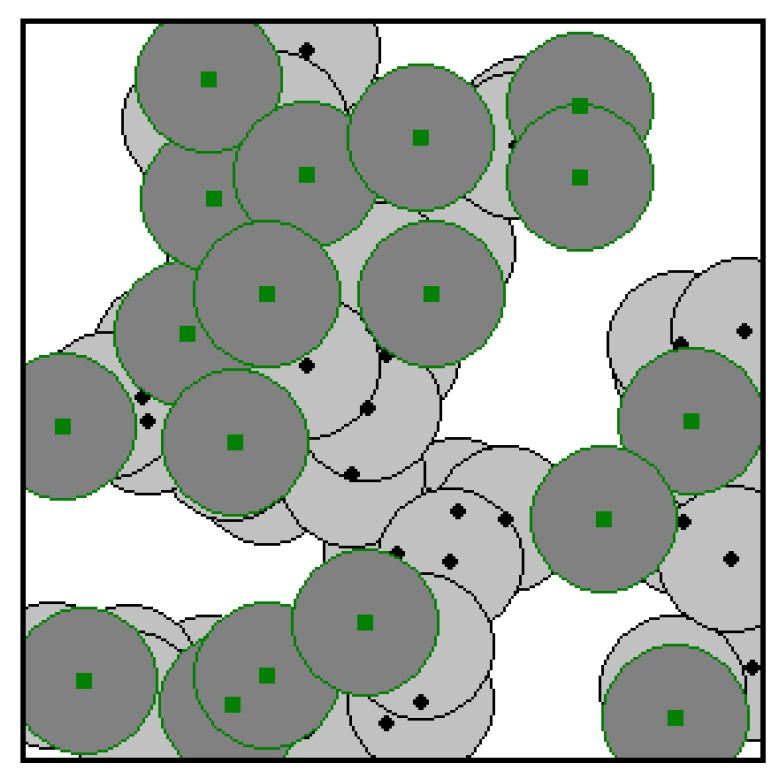
Initial deployment of the mobile sensor nodes.

In order to investigate the validity and efficiency of the proposed two-phase coverage-enhancing algorithm, we conducted different experiments to assess its performance. The simulation is implemented on an Intel(R) Core(TM) i3-4005U (1.7 GHz) computer using Delphi 6.0. The experiment used 60 randomly distributed sensor nodes within a 100 m × 100 m target area, among which 30% are mobile sensors. The initial distributions of all sensor nodes are shown in Figure 4. The sensing range is 10 m. The dark grey circles are the sensing areas of mobile sensors and the light grey circles are the sensing areas of static sensors. The small black solid circles represent static sensor nodes; while the small green solid rectangles represent the mobile sensor nodes. The maximum iteration number *T* is set to 1000, the population size is set to 10, the scaling factor *F* in Equation (9) is set to 0.6, and the crossover probability *CR* in Equation (10) is set to 0.95. It can be seen from Figure 4 that the target area has coverage holes and the sensing areas of some sensors are overlapping. The current area coverage rate of the hybrid WSN is 70%.

**Figure 5:**
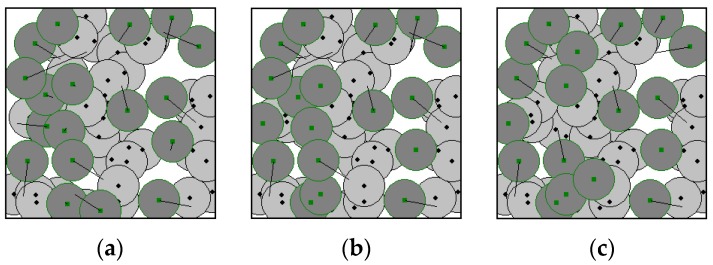
(**a**) Result of the proposed CEADE algorithm; (**b**) refinement result of Algorithm 1 step 1; (**c**) final refinement result.

Figure 5a–c shows the deployment results of our algorithm for the hybrid WSN shown in Figure 4. The solid lines represent the distance between the initial and the candidate target positions of each sensor nodes. The longer the line, the longer distance the mobile sensors have to move, resulting in larger energy consumption. Figure 5a shows the result of the proposed CEADE algorithm, where some mobile sensor nodes move to the coverage holes and the overall level of the overlapping sensing areas reduces, essentially increases the network coverage rate to 96%. As can be seen from Figure 5a, although all the mobile sensor nodes need to move, the moving distance varies between sensors, resulting in an average moving distance of 32.56 m. Figure 5b shows the result of refinement after step 1 of Algorithm 1, and it can easily be seen that the number of mobile sensor nodes that need to move reduces greatly. The moving-distance reduction optimization scheme in step 1 of Algorithm 1 eliminates the mobile sensor nodes that do not need to move, and results in having only 11 mobile sensors that need to move. The new average moving distance of mobile sensors is significantly reduced to 26.27 m. Figure 5c shows the final result of refinement, which is also the final result of the proposed two-phase algorithm. We can see from Figure 5c that the longer lines disappear, meaning that the moves with large moving distances are eliminated, resulting in an average moving distance of 22.04 m, which is shorter than that in Figure 5b. According to Figure 5, the average moving distance of mobile sensors reduces significantly after phase two.

**Figure 6:**
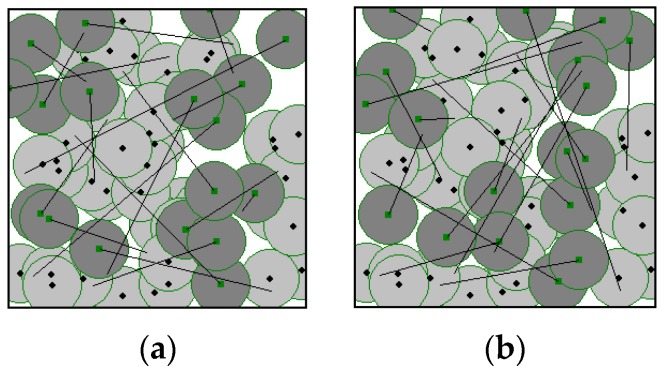
Mobile sensor node relocation results of (**a**) genetic algorithm (GA) in [24]; and (**b**) particle swarm optimization (PSO) in [25].

Figure 6a,b show the results of GA in [24] and PSO in [25], respectively, on the hybrid WSN as described in Figure 4 for comparison. Both GA and PSO achieve a network coverage rate of 99%, which is a little higher than that of our algorithm, however, the moving distance represented by the straight lines in Figure 6 are much longer than those in Figure 5c. The average moving distance of GA and PSO are 126.79 m and 137.36 m, respectively. That is to say, the average moving distance of the two algorithms is much longer than that of our algorithm, which means a higher moving cost as well as larger energy consumption are needed in the mobile sensor nodes. Both GA and PSO result in a longer average moving distance because the objective functions of GA in [24] and PSO in [25] do not take into account of the moving distance of the mobile sensor nodes. Thus, the objective functions can only guide the algorithms to search for solutions with a high area coverage rate but not one with a short moving distance, which leads to a long moving distance in both algorithms. 

**Figure 7:**
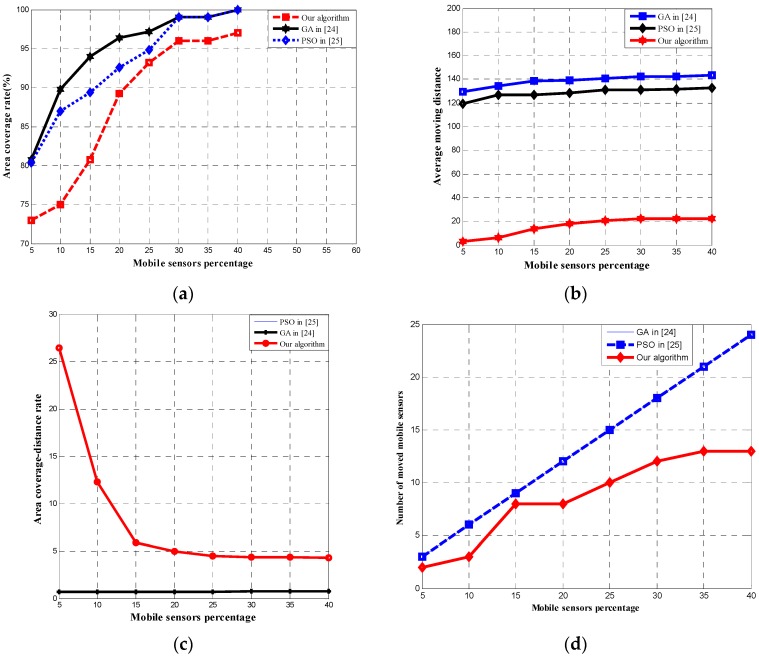
Results of the three algorithms on hybrid wireless sensor networks (WSNs) with different mobile sensors percentage. (**a**) Area coverage rate of the three algorithms; (**b**) average moving distance of the three algorithms; (**c**) area coverage–distance rate of the three algorithms; (**d**) the number of moved mobile sensors of the three algorithms.

In order to investigate the effect of the number of mobile sensor nodes to the area coverage rate, average moving distance, area coverage–distance rate, and the number of moved mobile sensors, we conducted a series of experiments on different hybrid WSNs which contain the same total number of sensor nodes but different number of mobile sensor nodes. The GA in [24], PSO in [25], and our algorithm are adopted to redeploy mobile sensor nodes to improve network coverage. Figure 7 shows the results of our study. For each data point in Figure 7, 30 trials were performed for each algorithm, and the results are averaged over the trials. As illustrated in Figure 7a, the area coverage rate of all the three algorithms increase with the mobile sensors percentage. Although the area coverage rate of the proposed algorithm is less than that of the other two algorithms, the area coverage is not the only metric for performance evaluation of coverage control algorithms. Figure 7b shows the average moving distance as a function of the of mobile sensors percentage achieved by the three algorithms. As shown in the figure, the average moving distance of our algorithm is significantly shorter than that of the other two algorithms, which means less energy consumption is required. The reason for this is that the moving distance of the mobile sensor nodes have not been taken into account in the objective functions of GA in [24] and PSO in [25], thus moving distance is not their optimization objective, resulting in a long moving distance in the two algorithms. On the other hand, our approach chose the area coverage–distance rate as the objective function of our DE algorithm, thus it can guide our algorithm to search for a solution with a high area coverage rate and a short moving distance. Therefore, the proposed algorithm can achieve a much shorter average moving distance than GA and PSO. Figure 7c shows that the area coverage–distance rate of GA in [24] is the same as that of PSO in [25], which is about 0.7. Furthermore, our algorithm has an area coverage–distance rate between 26.45 and 4.32, which is significantly better than the other two approaches. In addition, it can be seen from Figure 7d that the number of moved mobile sensors in [24] is also same as that of PSO in [25] because all the mobile sensors need to move to the candidate location. On the other hand, only a portion of the mobile sensor nodes need to move in our algorithm, resulting in a significantly smaller number of the moved mobile sensors in our algorithm.

The performance of most intelligent algorithms deteriorates rapidly as the dimensionality of the search space increases [31], which increases exponentially with the problem size. To investigate the effect of the number of wireless sensor nodes to the optimization performance, a series of experiments in different hybrid WSNs with different numbers of sensor nodes are used to test against various algorithm including the proposed two-phase algorithm, GA in [24], and PSO in [25]. The parameters of different hybrid WSNs are listed in Table 1.

**Figure 8:**
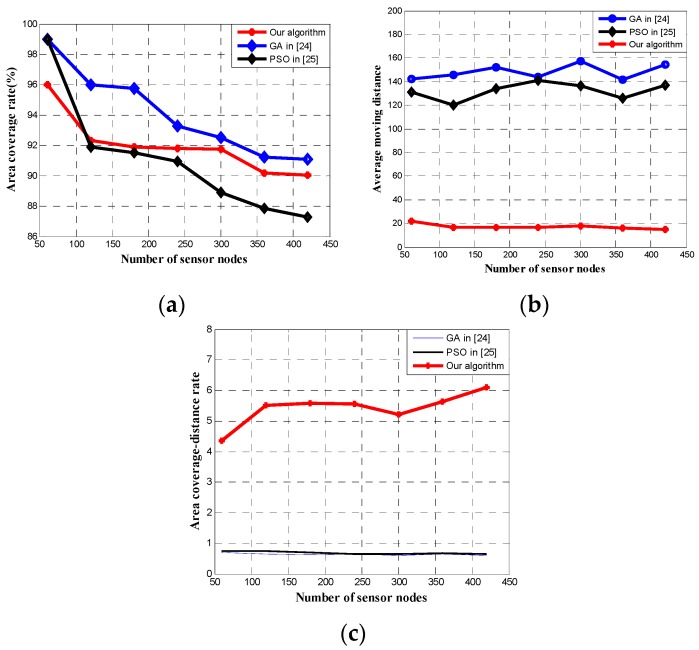
Results of the three algorithms on hybrid WSNs with different number of sensor nodes. (**a**) Area coverage rate of the three algorithms; (**b**) average moving distance of the three algorithms; (**c**) area coverage–distance rate of the three algorithms.

The GA in [24], PSO in [25], and our algorithms are adopted to deploy the sensor nodes in 50 independent operations for each WSN, respectively. The experimental results are shown in Figure 8. In Figure 8a, the area coverage rate of all the three algorithms decrease when the number of sensor nodes increases. The reason behind is that the performance of GA in [24], PSO in [25], and our DE algorithm will deteriorate as the number of wireless sensor nodes increases. The performance of the PSO deteriorates fastest among the three algorithms. When the number of sensor nodes reaches 120, the area coverage rate of PSO in [25] is less than that of our algorithm. Figure 8b shows the average moving distance against different number of sensor nodes in hybrid WSNs, resulted from each of the three algorithms that we are comparing. As shown, the average moving distance of our algorithm is much shorter than that of the other two algorithms, due to the well-designed objective function and the optimization scheme for moving distance reduction in our algorithm that involve both area coverage rate and moving distance of mobile sensor nodes. Figure 8c shows the area coverage–distance rate of the three algorithms on hybrid WSNs with different number of sensor nodes. Similar to Figure 7c, the area coverage–distance rate of our algorithm is much better than that of the other two algorithms, which is contributed from the objection function.

The comparison results of area coverage rate, average moving distance, area coverage–distance rate, and the number of the moved mobile sensors present the outstanding performance of the proposed two-phase coverage-enhancing algorithm for hybrid wireless sensor networks. The results demonstrate that our algorithm can achieve the best balance between the network coverage and the network energy consumption of moving mobile sensors. 

## 5. Conclusions

Redeployment of mobile sensor nodes improves network coverage in hybrid wireless sensor networks that consists of both mobile sensor nodes and stationary sensors nodes. In this paper, we propose a two-phase coverage-enhancing algorithm for hybrid WSNs. During the first phase, a differential evolution algorithm is adopted to compute the candidate target positions of mobile sensor nodes. During the second phase of refinement, an optimization scheme for moving-distance reduction is applied to the candidate target positions resulted from phase one, such that the mobile sensor nodes that are needed to move and their final target positions can be determined. Experimental results show that our algorithm can achieve better performance with respect to area coverage rate, average moving distance, area coverage–distance rate, and the number of moved mobile sensors, when compare with GA in [24] and PSO in [25]. The proposed coverage optimization algorithm can achieve a balance between network coverage and network energy consumption during mobile sensors relocation, as well as capable of redeploying mobile sensor nodes in hybrid wireless sensor networks effectively.

## Figures and Tables

**Table 1:** The parameters of different hybrid WSNs.

Number of Mobile Sensor Nodes	18	36	54	72	90	108	126
Number of static sensor nodes	42	84	126	168	210	252	294
Sensing range (m)	10	7	5.7	4.9	4.4	4	3.7
Communication range (m)	20	14	11.4	9.8	8.8	8	7.4
